# Food insecurity is inversely associated with positive childhood experiences among a nationally representative sample of children aged 0–17 years in the USA

**DOI:** 10.1017/S136898002300143X

**Published:** 2023-11

**Authors:** Xing Zhang, Meg Bruening, Chinedum O Ojinnaka

**Affiliations:** 1 College of Health Solutions, Arizona State University, Phoenix, AZ 85004, USA; 2 Department of Nutritional Sciences, Penn Stata College of Health and Human Development, University Park, PA, USA

**Keywords:** Positive childhood experiences, Food insecurity, Adverse childhood experiences, Life course, Children, Adolescents

## Abstract

**Objective:**

We examined the association between food insecurity and positive childhood experiences (PCE).

**Design:**

Outcome measure was number of PCE and seven PCE constructs. Food insecurity was assessed with a three-category measure that ascertained whether the respondent could afford and choose to eat nutritious food. We then used bivariate and multivariable Poisson and logistic regressions to analyse the relationship between food insecurity and the outcome measures. The analyses were further stratified by age (≤ 5, 6–11 and 12–17 years).

**Setting:**

The National Survey of Children’s Health (NSCH) from 2017 to 2020, a nationally representative sample of children and adolescents in the USA.

**Participants:**

Parents/caregivers who reported on their children’s experiences of PCE and food insecurity from the 2017–2020 NSCH (*n* 114 709).

**Results:**

Descriptively, 22·13 % of respondents reported mild food insecurity, while 3·45 % of respondents reported moderate to severe food insecurity. On multivariable Poisson regression analyses, there was a lower rate of PCE among children who experienced mild (incidence rate ratio (IRR) = 0·93; 95 % CI 0·92, 0·94) or moderate/severe food insecurity (IRR = 0·84; 95 % CI 0·83, 0·86) compared with those who were food secure. We found an inverse relationship between food insecurity and rate of PCE across all age categories.

**Conclusions:**

Our study finding lends evidence to support that interventions, public health programmes, as well as public health policies that reduce food insecurity among children and adolescents may be associated with an increase in PCE. Longitudinal and intervention research are needed to examine the mechanistic relationship between food insecurity and PCE across the life course.

Many young people experience some form of trauma that predicts health and nutrition-related behaviours and outcomes. Adverse childhood experiences (ACE) are traumatic events that occur during childhood (aged 0–17 years) that have negative consequences on an individual’s mental, physical, social and economic well-being^(1,2)^. Derived from the Centers for Disease Control-Kaiser ACE Study^(2)^, they include whether an individual has experienced divorce, had a family member go to prison, domestic violence (including verbal, physical and sexual abuse), neighbourhood violence, family depression and/or mental illness, family addiction to alcohol and drugs and discrimination. Approximately 25 % of individuals in the USA have reported experiencing at least three ACE^(3)^. By race and ethnicity, there is a higher prevalence of ACE among Black, Hispanic and multiracial individuals in the USA^(3)^. By socio-economic status, those who are unemployed, make less than $15 000 and had less than a high school education were more likely to report ACE^(3)^. Previous research has found that there is a direct relationship between ACE and food insecurity^(4,5)^, with greater ACE associated with a higher likelihood of food insecurity^(4–6)^.

While trauma-informed care and programming are critical to develop^(7)^, just as critical is understanding how resilience and asset-based approaches can promote positive health outcomes^(8)^. Positive childhood experiences (PCE), or benevolent childhood experiences or advantageous childhood experiences (counter-ACE), occur in children before age 18 and are beneficial to emotional and social development and well-being^(9)^. PCE can include having adults that children can rely on for advice or mentorship, having adults with whom to share ideas, living in a resilient household, living in a supportive neighbourhood, living in a safe neighbourhood and participating in social engagement through after-school activities and/or community service. PCE vary by socio-demographic characteristics. Rural–urban differences were observed in the reports of PCE, where rural youth were more likely to report volunteering and having a mentor as compared with urban youth^(10)^. Youth of colour are less likely to report living in a safe or supportive neighbourhood or having mentorship as compared with non-Hispanic White youth^(11)^. PCE may be associated with nutrition-related outcomes such as food insecurity; however, very little research has explored this association.

PCE have been shown to be a marker of resilience in risk behaviours and health outcomes for young people, independent of ACE. For example, Baglivio and Wolff reported that high PCE scores (more than 6) were related to lower recidivism rates for juvenile offenders, even after adjusting for ACE^(12)^. A retrospective study of adults found that when ACE were accounted for, PCE were related to families’ social and emotional health processes, families’ healthy lifestyles, families’ health resources and families’ external social support^(13)^. Further, research indicates that PCE moderate the relationship between ACE and health outcomes such as depression and anxiety^(14)^. Research has also shown that youth who were overweight or obese reported significantly fewer PCE than those who were underweight or normal weight; this relationship only held for those youth who reported two or more ACE^(15)^. However, whether there is a relationship between food insecurity and PCE is unknown, especially in the context of ACE. We expect that food insecurity may be associated with lower PCE, and that greater ACE may further exacerbate the negative association between food insecurity and PCE.

## Theoretical framework: life course and the stress process model

The study draws from two theoretical frameworks to examine the relationship between food insecurity and PCE: the life course framework^(16)^ and the stress process model^(17)^.

The life course framework ‘insists that development is lifelong and that no life stage can be understood in isolation from others’^(18)^. The developmental stages that we examine in this article include early childhood (aged 0–5 years), childhood and early adolescence (aged 6–11 years) and adolescence (aged 12–17 years). Three key concepts from the life course framework include *individual agency, linked lives* and *timing of events*. The concept of individual agency is that individuals can choose the activities they participate in, as well as relationships they develop, as they age. Another key concept of the life course framework is linked lives, or that children’s lives are interdependent with their families, neighbours, friends, students and community members. For example, food insecurity at the household level may affect the ability to cultivate nurturing and supportive relationships due to increased levels of stress, depression and anxiety^(19)^, thus impacting multiple family members living in the household, including parents and children. A third key concept is the importance of timing of events that occur in people’s lives. A disruptive and stressful event, such as experiencing food insecurity during infancy, may have consequences for a child’s likelihood of reporting ACE and PCE.

Food insecurity may also be related to ACE and PCE through the stress process model^(17)^. The stress process model states that stressful events, such as experiencing food insecurity, create role strain, making it difficult to effectively conduct one’s roles in life, such as parents/caregivers being able to provide adequate resources for children’s growth and development. In turn, the stressful event of experiencing food insecurity could decrease a person’s ability to develop mastery of skills and have positive self-esteem, which may lead to worse health outcomes, not only among parents but children themselves. This can manifest in a lower likelihood of children being able to experience PCE, such as having supportive relationships, living in safe environments and being socially engaged.

In addition, prior research has suggested that food insecurity has been associated with an increased likelihood of reporting ACE^(5)^. Food insecurity has been associated with worse physical and mental health outcomes, such as depression, delayed brain development, obesity and increased poverty, which have been linked to an increased likelihood of reporting ACE^(5,20)^.

However, it is unknown whether food insecurity is associated with PCE. As such, we examined whether there is an association between food insecurity and PCE. Given the literature on ACE discussed above, we hypothesised an inverse relationship between food insecurity and PCE after controlling for covariates including ACE. We also sought to examine how the associations vary by the age of the child during key stages in the life course.

To examine this question, we stratified results by early childhood (0–5 years), childhood and early adolescence (6–11 years) and adolescence (12–17 years). We expect that older children may have increased independence and autonomy from their parents as they progress through schooling and participate in greater activities in schools and neighbourhoods. Therefore, as children age, food insecurity may be associated with greater PCE among older children and food insecurity may be associated with lower PCE among younger children.

## Methods

### Data source

We use cross-sectional, publicly available data from the 2017–2020 National Survey of Children’s Health (NSCH). The NSCH is a multistage probability sampling survey funded by the US Health Resources and Services Administration Maternal and Child Health Bureau and administered by the Data Resource Center (https://www.childhealthdata.org/learn-about-the-nsch/NSCH). The Data Resource Center is supported by three US agencies: the Maternal and Child Health Bureau, the Health Resources and Services Administration and the US Department of Health and Human Services^(21)^. For academic research purposes, public use data can be requested from the following website: https://www.childhealthdata.org/dataset. The NSCH data provide information on several intersecting domains of children’s health including the child’s family, neighbourhood, school and social context. The survey is restricted to children aged 0–17 years. Respondents to the survey items are parents or caregivers but are framed in the viewpoint of the child, as recommended by NSCH^(22)^. We restricted the analytic sample to parents or caregivers who had complete information on measures and covariates used in the study. The final sample size was 114 709 parents or caregivers.

### Measures

#### Outcome variables: positive childhood experiences

The PCE were measured as a count variable (the total number of PCE) and in dichotomous forms based on seven constructs used by Crouch and colleagues^(10)^. The same outcome variables was used for the full sample (aged 0–17 years) and for analyses stratified by age groups.

We derived the total number of PCE by summing the number of PCE identified by a given respondent. Responses of ‘yes’ to binary variables were regarded as a PCE. Responses of all of the time, most of the time, some of the time, definitely agree or somewhat agree were also coded as a PCE.

Using their Health Outcomes from Positive Experiences framework, Sege and Brown identified four PCE constructs as follows: (1) ‘being in nurturing, supportive relationships’, (2) ‘living, developing, playing and learning in safe, stable, and equitable environments,’ (3) ‘having opportunities for constructive social engagement and to develop a sense of connectedness’ and (4) ‘learning of social and emotional competencies’. Crouch and colleagues operationalised these four constructs using variables that explored positive experiences in the NSCH data^(1–3)^.

Crouch and colleagues divided the first construct, *nurturing and supportive relationships*, into two additional constructs: mentorship and family resilience. *Mentorship* was operationalised using the question ‘other than you or other adults in your home, is there at least one adult in this child’s school, neighborhood, or community who knows this child well and who he or she can rely on for advice or guidance?’ *Family resilience* was operationalised using a composite measure derived from four questions: ‘when your family faces problems, how often are you likely to…? (1) talk together about what to do, (2) work together to solve our problems, (3) know we have strengths to draw on, and (4) stay hopeful even in difficult times’. The response options were all of the time, most of the time, some of the time and none of the time. The child was classified as residing in a household with family resilience if the caregiver responded all or most of the time to all four questions.

The second construct, *living in a stable, safe and equitable environment*, was also further divided into two constructs: *living in a supportive neighbourhood* and *living in a safe neighbourhood.* These two constructs were derived from the following question with four subitems: ‘To what extent do you agree with these statements about your neighborhood or community? (1) People in this neighborhood help each other out, (2) We watch out for each other’s children in this neighborhood, (3) This child is safe in our neighborhood, (4) When we encounter difficulties, we know where to go for help in our community’. The response options were definitely agree, somewhat agree, definitely disagree and disagree. *Living in a supportive neighbourhood* was operationalised using the following three items: ‘(1) People in this neighborhood help each other out, (2) We watch out for each other’s children in this neighborhood, and (3) When we encounter difficulties, we know where to go for help in our community’. Children were categorised as living in a supportive environment if the parent/caregiver gave a response of ‘definitely agree’ to at least 1 of the three questions, and ‘definitely agree’ or ‘somewhat agree’ to 2 other questions. *Living in a safe neighbourhood* was operationalised using the item ‘The child is safe in our neighbourhood’. The child was categorised as living in a safe neighbourhood if the caregiver responded with ‘definitely agree.’

The third construct, *opportunities for positive social engagement*, was also divided into two constructs: *social engagement that involves after-school activities* and *social engagement involving community service*. Social engagement involving after-school activities was operationalised using the following three questions: ‘During the past twelve months, did this child participate in a sports team or did he or she take sports lessons after school or on weekends? Any clubs or organizations after school or on weekends? Any other organized activities or lessons, such as music, dance, language, or arts?’ The variables were binary variables. The child was categorised as engaging in after-school social activities if the parent/caregiver provided a response of ‘yes’ to at least one of the questions. Social engagement involving community service was measured using the following question: ‘During the past twelve months did the child participate in any type of community service or volunteer work at school, place of worship, or in the community?’ A ‘yes’ response was categorised as participation in social engagement involving community service.

The final construct, *developing social and emotional competencies*, was measured using the question: ‘how well can you and this child share ideas or talk about things that really matter?’ Responses included very well, somewhat well, not very well or not very well at all. Responses of ‘very well’ or ‘somewhat well’ were regarded as developing social and emotional competencies while ‘not very well’ or ‘not very well at all’ was regarded as lack of development of social or emotional competencies.

#### Primary independent variable: food security status

The primary independent variable was food security status at the household level, which was measured using the following question adapted from the gold standard of measuring food security status in the USA from Module 24 of the USDA Household Food Security Survey^(23)^: ‘Which of these statements best describes the food situation in your household in the past 12 months?’ Response options were (1) We could always afford to eat good nutritious meals, (2) We could always afford enough to eat but not always the kinds of food we should eat, (3) Sometimes we could not afford enough to eat and (4) Often we could not afford enough to eat. Respondents who indicated that they could always afford to eat good nutritious meals were regarded as *food secure*. Respondents who could always afford enough to eat but not always the kinds of food they should eat were regarded as experiencing *mild food insecurity*. Respondents that indicated that they sometimes could not afford enough to eat or often could not afford enough to eat were regarded as experiencing *moderate to severe food insecurity*.

#### Covariates

Covariates included factors related to experiencing food insecurity, including the parent/caregiver’s educational attainment (less than high school, high school/vocational trade school and more than high school)^(24)^, the child’s age (0–5, 6–11 years old and 12–17 years), nativity (whether the respondent was born in the USA or not), race/ethnicity (White, Black/African American, Asian, Hispanic and Other), federal poverty level (0–99 %, 100–199 %, 200–349 % and ≥ 350 %), Supplemental Nutrition Assistance Program participation (yes, no), cash assistance (yes, no), receipt of free meals (yes, no), child’s special needs status (yes, no), general health (good/fair/poor health and excellent/very good health) and the number of ACE^(24)^. Nine questions were used to assess the number of ACE, a measure that has been previously validated^(25)^: (1) the family found it hard to cover basic needs like food or housing, whether the child was (2) a victim of violence or (3) treated unfairly because of their race, experienced (4) parent/guardian divorce, (5) death or (6) jail time, (7) an adult who physically assaulted others, (8) was mentally ill or (9) had alcohol problems. Federal poverty levels were derived as the ratio of total family income to the US Census Bureau’s family poverty threshold by NCSH and categorised as 0–99 %, 100–199 %, 200–349 % and 350 % and greater of the family poverty threshold^(22)^. Year fixed effects were used to control for potential temporal changes.

### Statistical analysis

We calculated summary statistics of the baseline characteristics of the study sample. We also conducted test of proportions using chi-square test. We then used bivariate and multivariable Poisson regression to analyse the relationship between food security status and the number of PCE. We report incidence rate ratios (IRR) and 95 % CI. The PCE score has a mean of 10·23 with variance of 7·52, suggesting overdispersion. However, sensitivity analyses conducted using negative binomial regression showed alpha values of approximately zero across all the models and the coefficients were consistent with the IRR from the Poisson regression. Bivariate and multivariable logistic regressions were used to analyse the relationship between food security status and the seven PCE constructs. We report OR and 95 % CI. The analyses were further stratified by age (≤ 5, 6–11 and 12–17 years). Sensitivity analyses were conducted controlling for BMI, which was ascertained for children who were 5 years or older; results were consistent with the main findings. This study was categorised as exempt from Human Subject Research oversight by the (blinded) Institutional Review Board. All analyses were adjusted for the NSCH complex survey design using sample weights and strata. Analyses were conducted using Stata 15^(21)^. Statistical significance was assessed at the *α* = 0·05 level.

## Results

### Sample characteristics

Table 1 shows summary statistics of the full sample and stratified by food security status. Among the full sample, most respondents reported being food secure (74·4 %). However, approximately 22·1 % of the respondents reported experiencing mild food insecurity, followed by 3·5 % who experienced moderate to severe food insecurity. A majority of caregivers had completed more than a high school degree (85·24 %), with 12·47 % completing high school or vocational trade school, and 2·30 % completing less than a high school degree. A majority of respondents (96·82 %) were born in the USA. By race, most respondents were White (68·92 %), followed by Hispanic (12·17 %), Other (7·88 %) and Asian (4·96 %). Most respondents did not participate in Supplemental Nutrition Assistance Program (89·16 %), receive cash assistance (97·30 %) or free meals (79·04 %). Most respondents reported that they had good, fair or poor health (92·08 %), followed by 7·92 % reporting that they had excellent or very good health. Approximately 23 % of parents/caregivers reported that their child had special needs. About 28 % of children were 5 years old and younger, 36 % were 6–11 years old and 35 % were 12–17 years old.

**Table 1:** Characteristics of respondents National Survey of Children’s Health, 2017–2020 (*n* 114 709)

	Full sample *n*	%	Food secure *n*	%	Mild food insecurity *n*	%	Moderate to severe food insecurity *n*	%	*P*
Household food security									
Food secure	85 365	74·42	–		–		–		
Mild food insecurity	25 389	22·13	–		–		–		
Moderate to severe food insecurity	3955	3·45	–		–		–		
Parent/caregiver’s educational attainment									< 0·0001
> High school	97 774	85·24	76 214	89·28	18 991	74·80	2569	64·96	
High school/vocational trade	14 300	12·47	7735	9·06	5439	21·42	1126	28·47	
< High school	2635	2·3	1416	1·66	959	3·78	260	6·57	
Born in the USA									< 0·0001
Yes	111 066	96·82	82 510	96·66	24 719	97·36	3837	97·02	
No	3643	3·18	2855	3·34	670	2·64	118	2·98	
Race/Ethnicity									< 0·0001
Non-Hispanic White	79 063	68·92	61 014	71·47	15 933	62·76	2116	53·50	
Non-Hispanic Black	6966	6·07	4201	4·92	2207	8·69	558	14·11	
Non-Hispanic Asian	5689	4·96	4774	5·59	825	3·25	90	2·28	
Hispanic	13 956	12·17	9153	10·72	4078	16·06	725	18·33	
Other	9035	7·88	6223	7·29	2346	9·24	466	11·78	
Federal poverty level (%)									< 0·0001
≥ 350	55 741	48·59	50 300	58·92	5206	20·50	235	5·94	
200–349	27 553	24·02	18 797	22·02	8053	31·72	703	17·77	
100–199	18 379	16·02	9715	11·38	7239	28·51	1425	36·03	
0–99	13 036	11·36	6553	7·68	4891	19·26	1592	40·25	
Supplemental Nutrition Assistance Program participation									< 0·0001
No	102 275	89·16	80 391	94·17	19 881	78·31	2003	50·64	
Yes	12 434	10·84	4974	5·83	5508	21·69	1952	49·36	
Cash assistance receipt									< 0·0001
No	111 610	97·3	83 832	98·20	24 189	95·27	3589	90·75	
Yes	3099	2·7	1533	1·80	1200	4·73	366	9·25	
Free meal receipt									< 0·0001
No	90 670	79·04	74 167	86·88	15 136	59·62	1367	34·56	
Yes	24 039	20·96	11 198	13·12	10 253	40·38	2588	65·44	
General health									< 0·0001
Excellent/very good	105 627	92·08	80 636	94·46	21 914	86·31	3077	77·80	
Good/fair/poor	9082	7·92	4729	5·54	3475	13·69	878	22·20	
Children with special needs									< 0·0001
No	88 025	76·74	67 564	79·15	18 066	71·16	2395	60·56	
Yes	26 684	23·26	17 801	20·85	7323	28·84	1560	39·44	
Age (years)									< 0·0001
≤ 5	32 625	28·44	24 975	29·26	6669	26·27	981	24·80	
6–11 years	41 578	36·25	30 425	35·64	9623	37·90	1530	38·69	
12–17 years	40 506	35·31	29 965	35·10	9097	35·83	1444	36·51	

Among those who were food secure, a higher proportion of parents/caregivers had more than a high school degree compared with parents who were high school/vocational trade graduates or who had less than a high school degree (89·28 % *v*. 9·06 % *v*. 1·66 %), respectively. About 72 % of food secure respondents were White compared with 5 %, 6 %, 11 % and 7 % of Black, Asian, Hispanic and ‘other’ respondents, respectively.

Among those who experienced moderate to severe food insecurity, a higher proportion of parents/caregivers had more than a high school degree compared with those who were high school graduates/vocational trade or who had less than a high school degree (64·96 % *v*. 28·47 % *v*. 6·57 %), respectively. About 53·50 % of respondents who experienced moderate to severe food insecurity were White parents/caregivers compared with 14 %, 2 %, 18 % and 12 % of Black, Asian, Hispanic and ‘other’ parents/caregivers, respectively.

### Regression analyses

Table 2 shows regression analyses of the association of PCE and food security status, among the full sample, and stratified by children’s age.

**Table 2:** Regression analyses of association between food security status and positive childhood experiences among respondents of the National Survey of Children’s Health, 2017–2020^†^

	Number of positive childhood experiences^‡^															
	Full sample (*n* 114 709)				≤ 5 years (*n* 32 625)				6–11 years (*n* 41 578)				12–17 years (*n* 40 506)			
	Unadjusted		Adjusted		Unadjusted		Adjusted		Unadjusted		Adjusted		Unadjusted		Adjusted	
	IRR	95 % CI	IRR	95 % CI	IRR	95 % CI	IRR	95 % CI	IRR	95 % CI	IRR	95 % CI	IRR	95 % CI	IRR	95 % CI
Food security status																
Food secure	Ref.		Ref.		Ref.		Ref.		Ref.		Ref.		Ref.		Ref.	
Mild food insecurity	0·93	0·92, 0·94^***^	0·96	0·95, 0·97^***^	0·94	0·93, 0·95^***^	0·96	0·95, 0·97^***^	0·91	0·90, 0·92^***^	0·96	0·95, 0·97^***^	0·91	0·90, 0·92^***^	0·96	0·95, 0·97^***^
Moderate to severe food insecurity	0·84	0·83, 0·86^***^	0·91	0·90, 0·93^***^	0·85	0·82, 0·88^***^	0·88	0·85, 0·91^***^	0·84	0·82, 0·86^***^	0·93	0·90, 0·95^***^	0·82	0·80, 0·85^***^	0·91	0·88, 0·94^***^
	Mentorship															
	Full sample (*n* 114 709)				≤ 5 years (*n* 32 625)				6–11 years (*n* 41 578)				12–17 years (*n* 40 506)			
	Unadjusted		Adjusted		Unadjusted		Adjusted		Unadjusted		Adjusted		Unadjusted		Adjusted	
	OR	95 % CI	OR	95 % CI	OR	95 % CI	OR	95 % CI	OR	95 % CI	OR	95 % CI	OR	95 % CI	OR	95 % CI
Food security status																
Food secure	Ref.		Ref.		Ref.		Ref.		Ref.		Ref.		Ref.		Ref.	
Mild food insecurity	0·59	0·52, 0·68^***^	0·73	0·63, 0·84^***^					0·57	0·48, 0·67^***^	0·68	0·57, 0·82^***^	0·63	0·51, 0·78^***^	0·79	0·62, 1·00
Moderate to severe food insecurity	0·39	0·31, 0·49^***^	0·53	0·41, 0·70^***^					0·41	0·32, 0·53^***^	0·53	0·38,0·74^***^	0·37	0·25, 0·55^***^	0·50	0·33, 0·77^***^
	Resilience															
	Full sample (*n* 114 709)				≤ 5 years (*n* 32 625)				6–11 years (*n* 41 578)				12–17 years (*n* 40 506)			
	Unadjusted		Adjusted		Unadjusted		Adjusted		Unadjusted		Adjusted		Unadjusted		Adjusted	
	OR	95 % CI	OR	95 % CI	OR	95 % CI	OR	95 % CI	OR	95 % CI	OR	95 % CI	OR	95 % CI	OR	95 % CI
Food security status																
Food secure	Ref.		Ref.		Ref.		Ref.		Ref.		Ref.		Ref.		Ref.	
Mild food insecurity	0·51	0·47, 0·55^***^	0·62	0·55, 0·70^***^	0·45	0·38, 0·53^***^	0·57	0·46, 0·70^***^	0·54	0·48, 0·62^***^	0·66	0·57, 0·77^***^	0·54	0·47,0·62^***^	0·70	0·60, 0·82^***^
Moderate to severe food insecurity	0·30	0·25, 0·34^***^	0·44	0·34, 0·58^***^	0·23	0·17, 0·31^***^	0·40	0·28, 0·57^***^	0·35	0·28, 0·44^***^	0·58	0·45, 0·74^***^	0·30	0·23, 0·39^***^	0·50	0·37, 0·68^***^
	Supportive neighbourhood															
	Full sample (*n* 114 709)				≤ 5 years (*n* 32 625)				6–11 years (*n* 41 578)				12–17 years (*n* 40 506)			
	Unadjusted		Adjusted		Unadjusted		Adjusted		Unadjusted		Adjusted		Unadjusted		Adjusted	
	OR	95 % CI	OR	95 % CI	OR	95 % CI	OR	95 % CI	OR	95 % CI	OR	95 % CI	OR	95 % CI	OR	95 % CI
Food security status																
Food secure	Ref.		Ref.		Ref.		Ref.		Ref.		Ref.		Ref.		Ref.	
Mild food insecurity	0·38	0·35, 0·40^***^	0·48	0·44, 0·51^***^	0·41	0·36, 0·46^***^	0·47	0·41, 0·53^***^	0·37	0·34, 0·41^***^	0·48	0·43, 0·54^***^	0·35	0·31, 0·39^***^	0·47	0·41, 0·53^***^
Moderate to severe food insecurity	0·25	0·22, 0·30^***^	0·41	0·35, 0·49^***^	0·27	0·19, 0·38^***^	0·37	0·26, 0·52^***^	0·27	0·22, 0·33^***^	0·45	0·35, 0·57^***^	0·22	0·18, 0·28^***^	0·40	0·30, 0·52^***^
	Safe neighbourhood															
	Full sample (*n* 114 709)				≤ 5 years (*n* 32 625)				6–11 years (*n* 41 578)				12–17 years (*n* 40 506)			
	Unadjusted		Adjusted		Unadjusted		Adjusted		Unadjusted		Adjusted		Unadjusted		Adjusted	
	OR	95 % CI	OR	95 % CI	OR	95 % CI	OR	95 % CI	OR	95 % CI	OR	95 % CI	OR	95 % CI	OR	95 % CI
Food security status																
Food secure	Ref.		Ref.		Ref.		Ref.		Ref.		Ref.		Ref.		Ref.	
Mild food insecurity	0·39	0·36, 0·41^***^	0·49	0·46, 0·53^***^	0·41	0·36, 0·46^***^	0·47	0·41, 0·53^***^	0·41	0·37, 0·45^***^	0·52	0·46, 0·59^***^	0·34	0·30, 0·38^***^	0·46	0·40, 0·52^***^
Moderate to severe food insecurity	0·26	0·23, 0·30^***^	0·42	0·36, 0·49^***^	0·23	0·17, 0·30^***^	0·30	0·22, 0·41^***^	0·28	0·23, 0·35^***^	0·45	0·36, 0·57^***^	0·27	0·21, 0·35^***^	0·50	0·38, 0·66^***^
	Social engagement involving after-school activity															
	Full sample (*n* 114 709)				≤ 5 years (*n* 32 625)				6–11 years (*n* 41 578)				12–17 years (*n* 40 506)			
	Unadjusted		Adjusted		Unadjusted		Adjusted		Unadjusted		Adjusted		Unadjusted		Adjusted	
	OR	95 % CI	OR	95 % CI	OR	95 % CI	OR	95 % CI	OR	95 % CI	OR	95 % CI	OR	95 % CI	OR	95 % CI
Food security status																
Food secure	Ref.		Ref.		Ref.		Ref.		Ref.		Ref.		Ref.		Ref.	
Mild food insecurity	0·48	0·44, 0·53^***^	0·78	0·70, 0·87^***^					0·45	0·39, 0·50^***^	0·75	0·65, 0·87^***^	0·54	0·47, 0·63^***^	0·81	0·69, 0·96^*^
Moderate to severe food insecurity	0·35	0·29, 0·42^***^	0·80	0·65, 0·97^*^					0·32	0·26, 0·40^***^	0·81	0·62, 1·05	0·40	0·30, 0·52^***^	0·79	0·59, 1·06
	Social engagement involving community service															
	Full sample (*n* 114 709)				≤ 5 years (*n* 32 625)				6–11 years (*n* 41 578)				12–17 years (*n* 40 506)			
	Unadjusted		Adjusted		Unadjusted		Adjusted		Unadjusted		Adjusted		Unadjusted		Adjusted	
	OR	95 % CI	OR.	95 % CI	OR	95 % CI	OR	95 % CI	OR	95 % CI	OR	95 % CI	OR	95 % CI	OR	95 % CI
Food security status																
Food secure	Ref.		Ref.		Ref.		Ref.		Ref.		Ref.		Ref.		Ref.	
Mild food insecurity	0·69	0·64, 0·74^***^	0·96	0·88, 1·04					0·75	0·67, 0·83^***^	1·02	0·91, 1·14	0·60	0·54, 0·67^***^	0·89	0·78, 1·01
Moderate to severe food insecurity	0·53	0·45, 0·63^***^	0·95	0·79, 1·16					0·59	0·48, 0·74^***^	1·03	0·80, 1·33	0·45	0·35, 0·59^***^	0·87	0·65, 1·16
	Sharing ideas															
	Full sample (*n* 114 709)				≤ 5 years (*n* 32 625)				6–11 years (*n* 41 578)				12–17 years (*n* 40 506)			
	Unadjusted		Adjusted		Unadjusted		Adjusted		Unadjusted		Adjusted		Unadjusted		Adjusted	
	OR	95 % CI	OR	95 % CI	OR	95 % CI	OR	95 % CI	OR	95 % CI	OR	95 % CI	OR	95 % CI	OR	95 % CI
Food security status																
Food secure	Ref.		Ref.		Ref.		Ref.		Ref.		Ref.		Ref.		Ref.	
Mild food insecurity	0·65	0·60, 0·70^***^	0·72	0·66, 0·79^***^					0·63	0·57, 0·70^***^	0·72	0·64, 0·80^***^	0·67	0·60, 0·75^***^	0·73	0·64, 0·83^***^
Moderate to severe food insecurity	0·72	0·62, 0·86^***^	0·99	0·82, 1·19					0·71	0·58, 0·88^***^	1·09	0·86, 1·38	0·73	0·57, 0·94^*^	0·88	0·66, 1·19

IRR, incidence rate ratio; Ref, reference category.

* Indicates *P*-value < 0·05.

*** Indicates *P*-value < 0·001.

† Adjusted for parent’s educational attainment, child’s age, nativity, race/ethnicity, federal poverty level, Supplemental Nutrition Assistance Program participation, cash assistance, receipt of free meal, general health, special needs status, number of adverse childhood experiences (ACE) and year of survey. Covariates are adjusted for the full sample and the stratified sample.

‡ Poisson regression is used for number of positive childhood experiences; all other outcomes were analysed using logistic regression.

### Regression analyses of food security status and number of positive childhood experience and positive childhood experience constructs among the full sample

#### Number of positive childhood experiences

On bivariate analyses, compared with those who were food secure, there was a statistically significant inverse relationship between mild food insecurity (IRR = 0·93; 95 % CI 0·92, 0·94) and moderate/severe food insecurity (IRR = 0·84; 95 % CI 0·83, 0·86) and the number of PCE. Adjusting for covariates, the statistically significant inverse relationship between mild (IRR = 0·96; 95 % CI 0·95, 0·97) or moderate food insecurity (IRR = 0·91; 95 % CI 0·90, 0·93) and the number of PCE persisted.

#### Positive childhood experience constructs

Overall, children who experienced mild or moderate/severe food insecurity had a significantly lower likelihood of having a mentor, experiencing family resilience, living in a supportive neighbourhood, living in a safe neighbourhood, participating in after-school activities, participating in community service and sharing ideas, in both bivariate analyses and after adjusting for covariates.

On bivariate analyses, there was a decreased likelihood of having a mentor among those who experienced mild (OR = 0·59; 95 % CI 0·52, 0·67) or moderate/severe food insecurity (OR = 0·39; 95 % CI 0·31, 0·49) compared with those who were food secure. This relationship persisted after adjusting for covariates, with a decreased likelihood of having a mentor among those who had experienced mild food insecurity (OR = 0·73; 95 % CI 0·63, 0·84) and those who had experienced moderate/severe food insecurity (OR = 0·53; 95 % CI 0·41, 0·70).

For analyses on family resilience, there was also a statistically significant association between food security status and resilience on bivariate analyses which persisted upon adjusting for covariates, with a decreased likelihood of resilience among those who experienced mild (OR = 0·62; 95 % CI 0·55, 0·70) or moderate/severe food insecurity (OR = 0·44; 95 % CI 0·34, 0·58), relative to those who were food secure.

For analyses on living in a supportive neighbourhood, there was also a statistically significant relationship between food security status and reporting a supportive neighbourhood on bivariate analyses. This association also persisted on multivariable analysis, with a decreased likelihood of reporting a supportive neighbourhood among those who experienced mild (OR = 0·48; 95 % CI 0·44, 0·51) or moderate/severe food insecurity (OR = 0·41; 95 % CI 0·35, 0·49) compared with those who were food secure.

For living in a safe neighbourhood, bivariate analyses showed a decreased likelihood of reporting a safe neighbourhood among those who experienced mild or moderate/severe food insecurity compared with those who were food secure. This relationship persisted on multivariable analysis, with a decreased likelihood of reporting a safe neighbourhood among those who experienced mild food (OR = 0·49; 95 % CI 0·46, 0·53) or moderate/severe food insecurity (OR = 0·42; 95 % CI 0·36, 0·49).

For participation in after-school activities, bivariate analyses indicated that there was a decreased likelihood of participating in after-school activities among those who experienced mild (OR = 0·48; 95 % CI 0·44, 0·53) or moderate food insecurity (OR = 0·35; 95 % CI 0·29, 0·42), relative to those who were food secure. This relationship persisted on multivariable regression with a decreased likelihood of engaging in after-school activities among those who experienced mild (OR = 0·78; 95 % CI 0·70, 0·87) or moderate/severe food insecurity (OR = 0·80; 95 % CI 0·65, 0·97) relative to those who were food secure.

Regarding community service, on bivariate analyses, there was also a decreased likelihood of community service among those who experienced mild (OR = 0·69; 95 % CI 0·64, 0·74) or moderate/food insecurity (OR = 0·53; 95 % CI 0·45, 0·63) relative to those who were food secure. However, this relationship was no longer statistically significant upon adjusting for covariates.

For analyses on sharing ideas, there was a decreased likelihood of sharing ideas with children among those who experienced mild (OR = 0·65; 95 % CI 0·60, 0·70) or moderate/severe food insecurity (OR = 0·72; 95 % CI 0·62, 0·86), relative to those who were food secure. Upon adjusting for covariates, there was a decreased likelihood of sharing ideas with children among those who experienced mild food insecurity (OR = 0·72; 95 % CI 0·66, 0·79). The relationship between sharing ideas and moderate/severe food insecurity was no longer statistically significant.

### Stratification analyses by age groups

The results of the stratification analyses by children’s age groups (0–5 years, 6–11 years old and 12–17 years old) were consistent with the full sample analyses (Table 2).

## Discussion

This study sought to examine the relationship between food security status and PCE among a nationally representative sample of children aged 0–17 years in the USA. To our knowledge, this is the first study that has examined this relationship. As expected, there was an inverse association between food insecurity and PCE, even after controlling for ACE. We were also interested to assess if there were differences in the relationship between food security status and PCE across the childhood life course. Surprisingly, we did not observe differences in associations of either the PCE as a whole or the constructs of PCE by childhood life stage. Across all children’s age categories, there was a statistically significant inverse relationship between food security status and PCE. These findings set the stage for future research using a resilience framework and asset-based approach to address public health child nutrition outcomes such as food insecurity.

Food insecurity is associated with many physical, social, emotional and environmental factors^(20,26–30)^. Given the persistent direct association between food insecurity and ACE, we expected and observed a statistically significant relationship between food insecurity and several constructs of PCE (mentorship resilience, supportive neighbourhood, safe neighbourhood, social engagement involving after-school activities and sharing ideas). This suggests that food insecure children may have fewer social and economic resources, which may be associated with a lower likelihood of experiencing PCE^(31)^. Safe and supportive neighbourhoods had the strongest inverse relationship with food insecurity (51–58 % lower odds of food insecurity). Environmental factors such as safe and supportive neighbourhoods have long been linked to health outcomes for children such as increased physical activity and lower alcohol and drug use^(32)^. Research has also indicated that neighbourhood poverty is a predictor of child, but not adult, food insecurity, when controlling for family factors (e.g. household poverty, parents’ marital status)^(33)^. In the current study, safe and supportive neighbourhoods may be a proxy for the overall environment in the neighbourhood, facilitating access to healthy, affordable foods. Families who report less safe and supportive neighbourhoods may travel greater distances for employment and have more limited time for food shopping and preparation. Future research should examine how systems-level environmental changes are linked to improvements in PCE and food insecurity for vulnerable children.

For most PCE, we observed a dose-dependent relationship – with higher food insecurity, there was a lower prevalence of PCE. This finding is supported by the literature that suggests some experiences with food insecurity (i.e. marginal food security as defined by the USDA) are closer in effect to experiences of low and very low food security than they are to food security^(34–37)^. Specifically, research indicates that adults with marginal food security have similar adverse health outcomes (e.g. dietary quality, mental health), to those with food insecurity. In fact, Cook *et al*. suggest that marginal food security should be considered food insecurity (which USDA classifies as low and very low food security)^(35)^. Study findings from the current study support this notion as mild food insecurity was associated with experiencing fewer PCE overall and a lower likelihood of having a mentor, living in a support and safe neighbourhood, experiencing family resilience, participating in after-school activities and community service and sharing ideas. Together with this study, the literature indicates that the prevention of any experiences with food insecurity is important for the health and development of children and adolescents.

Our study is the first to examine how the relationship between food insecurity and PCE varies across children’s age group. We examined this relationship among children aged 0–5 years old, 6–11 years old and 12–17 years old, capturing the developmental periods of early childhood, middle childhood and adolescence. Across all age groups, food insecurity was associated with a decreased likelihood of reporting PCE. Therefore, food insecurity may be a form of trauma that is associated with decreased PCE, regardless of age or timing across the life course. We did not find support to lend the idea that as children grew older, they would have greater individual agency, as well as autonomy^(38)^, and seek out more PCE regardless of food security status. Supporting the life course framework concept of linked lives, or that the experiences of a parent or caregiver can affect the experiences of a child and vice versa throughout the life course, food insecurity has been associated with negative outcomes related to nutrition, mental health, physical health and economic outcomes among parents and family caregivers^(16,39)^. In the current study, we found that a parent/caregiver’s report of household food insecurity was associated with lower reports of PCE among their children, which is likely a reflection of parent/caregiver’s context and experiences. More research is needed to understand the interplay between parents’ reports and children’s experiences, as the spillover effects of PCE and food insecurity may vary by household. In sum, our results demonstrate that it is important to consider interventions and public policies that reduce food insecurity at all ages across the lifespan, as it may be associated with negative outcomes for children’s PCE across the lifespan.

### Strengths and limitations

There are several strengths and limitations that should be taken into consideration when examining the findings. This study used a large, nationally representative sample of families in the USA, which allowed us to examine differences in the relationship between food security status and PCE across the life course. Parents/caregivers reported on children’s PCE and food insecurity, which may be prone to social desirability bias. Only one item was used to measure food insecurity, which may overestimate and underestimate the prevalence of food insecurity^(40)^. While the NCHS survey used an adaptation of a validated food insecurity tool from the USDA Household Food Security survey, this particular version has yet to be validated. However, this measure has been widely used and is theoretically grounded. It is also important to note that all items on the NCHS go through a rigorous inclusion process by the US Maternal Child Health Bureau. Finally, these were cross-sectional data; as such, causality cannot be inferred.

## Conclusion

Using nationally representative data in the USA, we found a dose–response relationship between food insecurity and PCE for children aged 0–17 years. This finding lends evidence to support that interventions, public health programmes, as well as public health policies that reduce food insecurity among children and adolescents may be associated with an increase in PCE in the USA. Examples of public health programmes and policies to reduce food insecurity in the USA include the Supplemental Nutrition Assistance Program, Supplemental Nutrition Assistance Program for Women, Infants and Children and the National School Lunch Program. Longitudinal and intervention research is needed to examine the mechanistic relationship of food insecurity and PCE across the life course.
